# Supplementary material for: Data heterogeneity in federated learning with Electronic Health Records: Case studies of risk prediction for acute kidney injury and sepsis diseases in critical care
Source: PLOS Digit Health. 2023 Mar 15;2(3):e0000117. doi: 10.1371/journal.pdig.0000117 (PMC10016691; doi:10.1371/journal.pdig.0000117)
Supplement: S3 Table — Performances of LR and MLP models are shown for each model framework. Four metrics are captured: accuracy, AUC, precision, and recall. (DOCX) [file pdig.0000117.s003.docx]

**
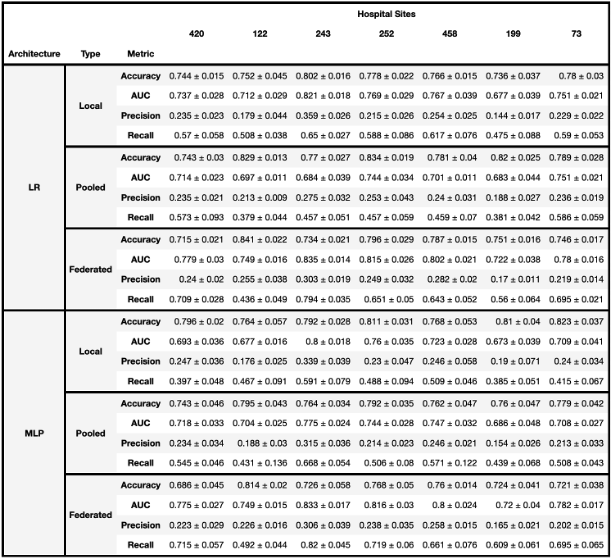
**

**S3 Table. Performance summaries of all AKI 48h models.** Performances of LR and MLP models are shown for each model framework. Four metrics are captured: accuracy, AUC, precision, and recall.
