# Supplementary figures and images for: Data heterogeneity in federated learning with Electronic Health Records: Case studies of risk prediction for acute kidney injury and sepsis diseases in critical care
Source: PLOS Digit Health. 2023 Mar 15;2(3):e0000117. doi: 10.1371/journal.pdig.0000117 (PMC10016691; doi:10.1371/journal.pdig.0000117)

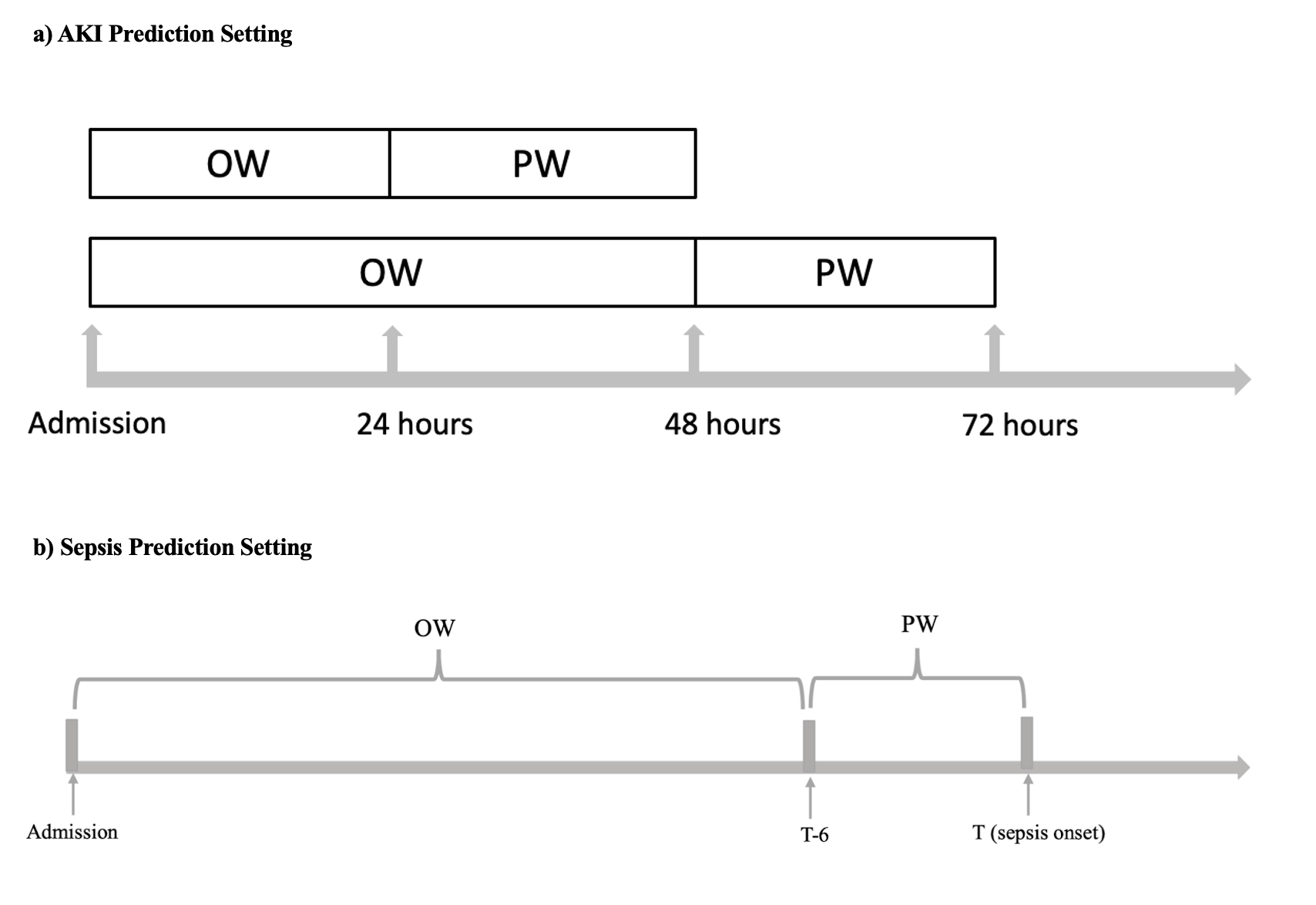

Supplement: S1 Fig — For AKI prediction, there are two observation windows (2 OWs) which creates 2 AKI prediction settings. The observation window for AKI settings can be 24 or 48 hours. For sepsis prediction, the observation window is the entire period from admission to 6 hours prior to the onset of sepsis. (TIF) [file pdig.0000117.s004.tif]

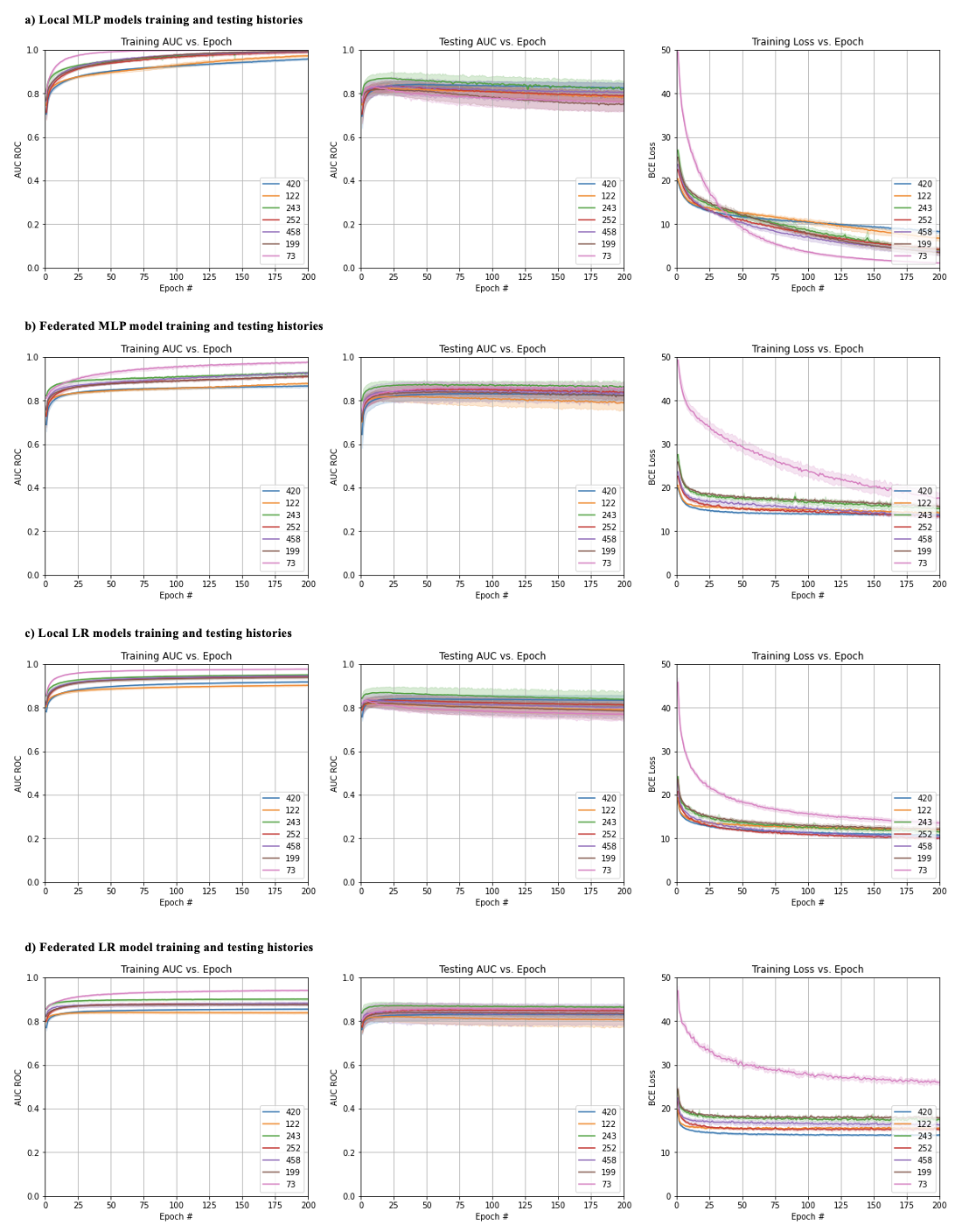

Supplement: S2 Fig — Training AUC, testing AUC, and training loss at each epoch (from left to right) has been shown. (a, c) show local histories where each color indicates the histories of a different site. (b, d) show histories for the federated model, where each color shows the history of the model while training/testing on that site’s data. Training and testing histories for AKI settings show similar patterns to the sepsis setting. (TIF) [file pdig.0000117.s005.tif]

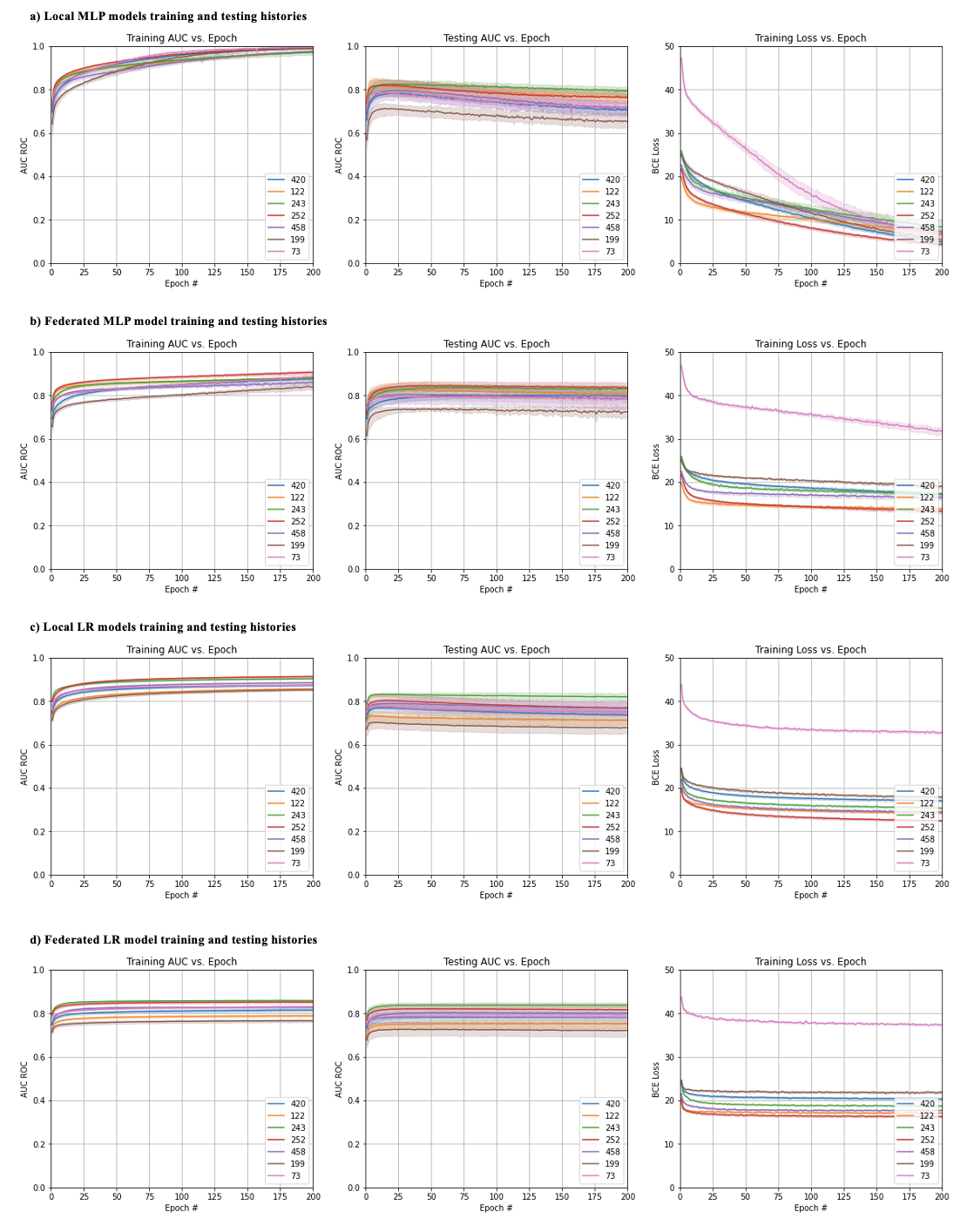

Supplement: S3 Fig — Training AUC, testing AUC, and training loss at each epoch (from left to right) has been shown. (a, c) show local histories where each color indicates the histories of a different site. (b, d) show histories for the federated model, where each color shows the history of the model while training/testing on that site’s data. Training and testing histories for AKI settings show similar patterns to the sepsis setting. (TIF) [file pdig.0000117.s006.tif]

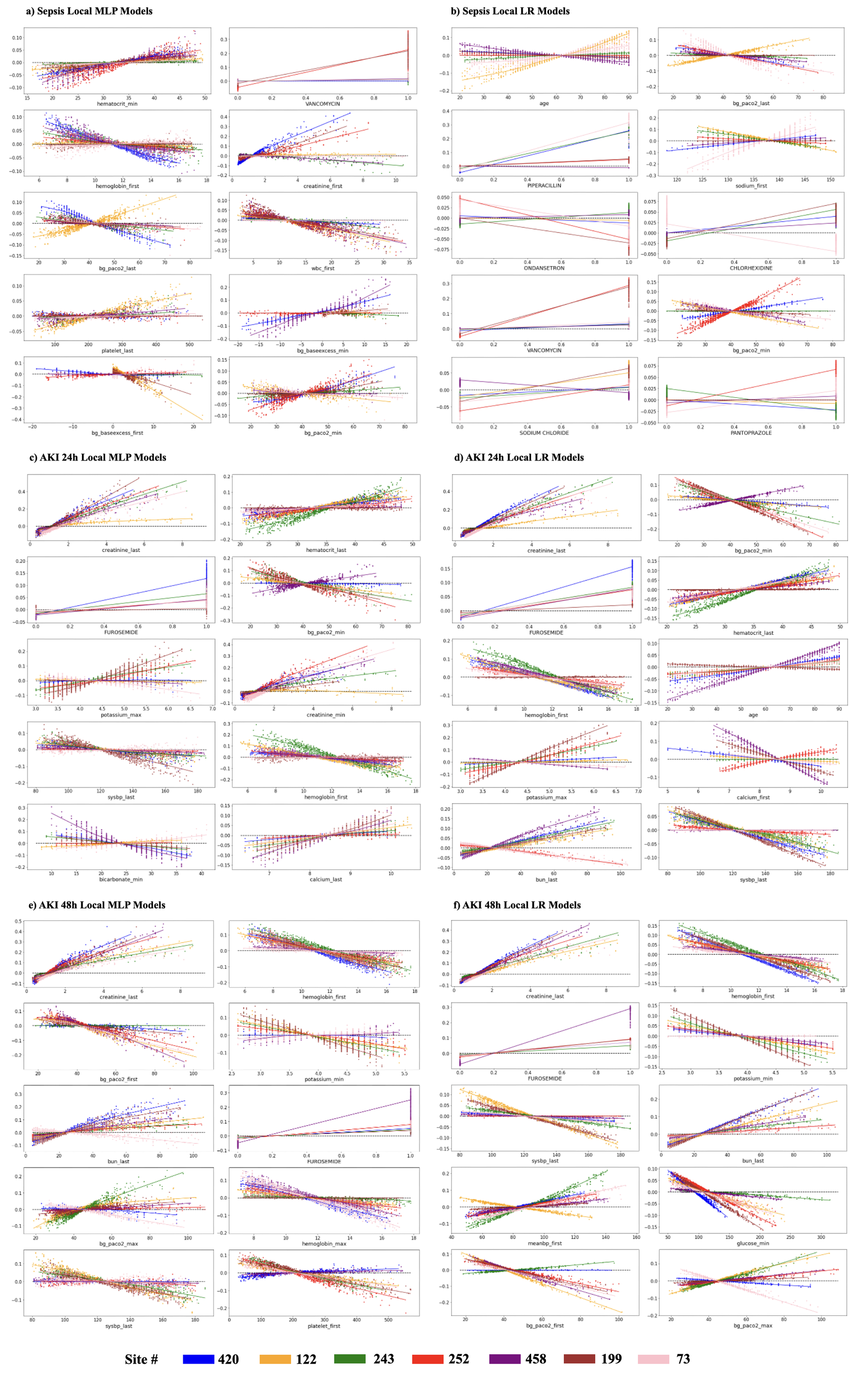

Supplement: S4 Fig — Each panel shows the marginal effects of impactful features for predicting sepsis, AKI 24h, or 48h in all local site models. All 7 sites are plotted in each panel, where each color corresponds to a different site (see legend). The x-axis gives the raw values of each feature, and the y-axis gives the logarithmic of estimated odds ratio (i.e., the SHAP value) for sepsis, AKI 24h or AKI 48h, when a feature takes a certain value. Each dot represents the SHAP value of a sample. The LOWESS curve, used for smoother extrapolating across all the dots, is plotted in all panels for each site. (a, c, and e) show Shapley dependence plots for federated MLP models and (b, d, and f) show Shapley dependence plots for federated LR models. (a, b) show plots for sepsis, (c, d) show plots for AKI 24h, and (e, f) show plots for AKI 48h. (TIF) [file pdig.0000117.s007.tif]

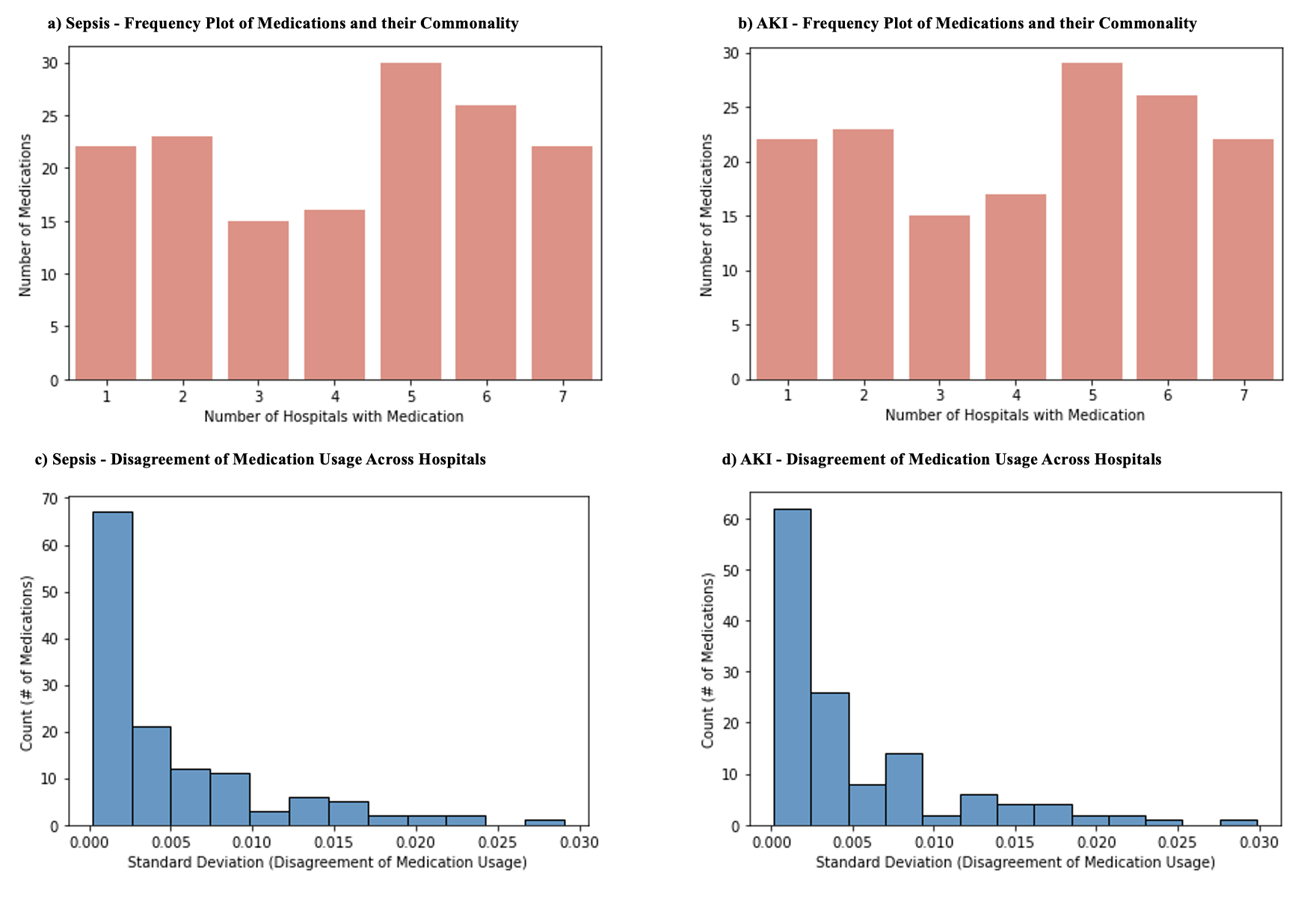

Supplement: S5 Fig — (a, b) shows frequency of medications across hospitals. X-axis is the number of hospitals and y-axis is the number of medications. For example, there are ~20 medications that only appear at 1 hospital. (c, d) show disagreement of medication usage across hospitals for medications that appear at 2 or more hospitals. X-axis shows the standard deviation bins of proportions of patients using the medication at each hospital (i.e., larger values of standard deviation indicate more disagreement). Y-axis shows the number of medications within the histogram bin. (TIF) [file pdig.0000117.s008.tif]

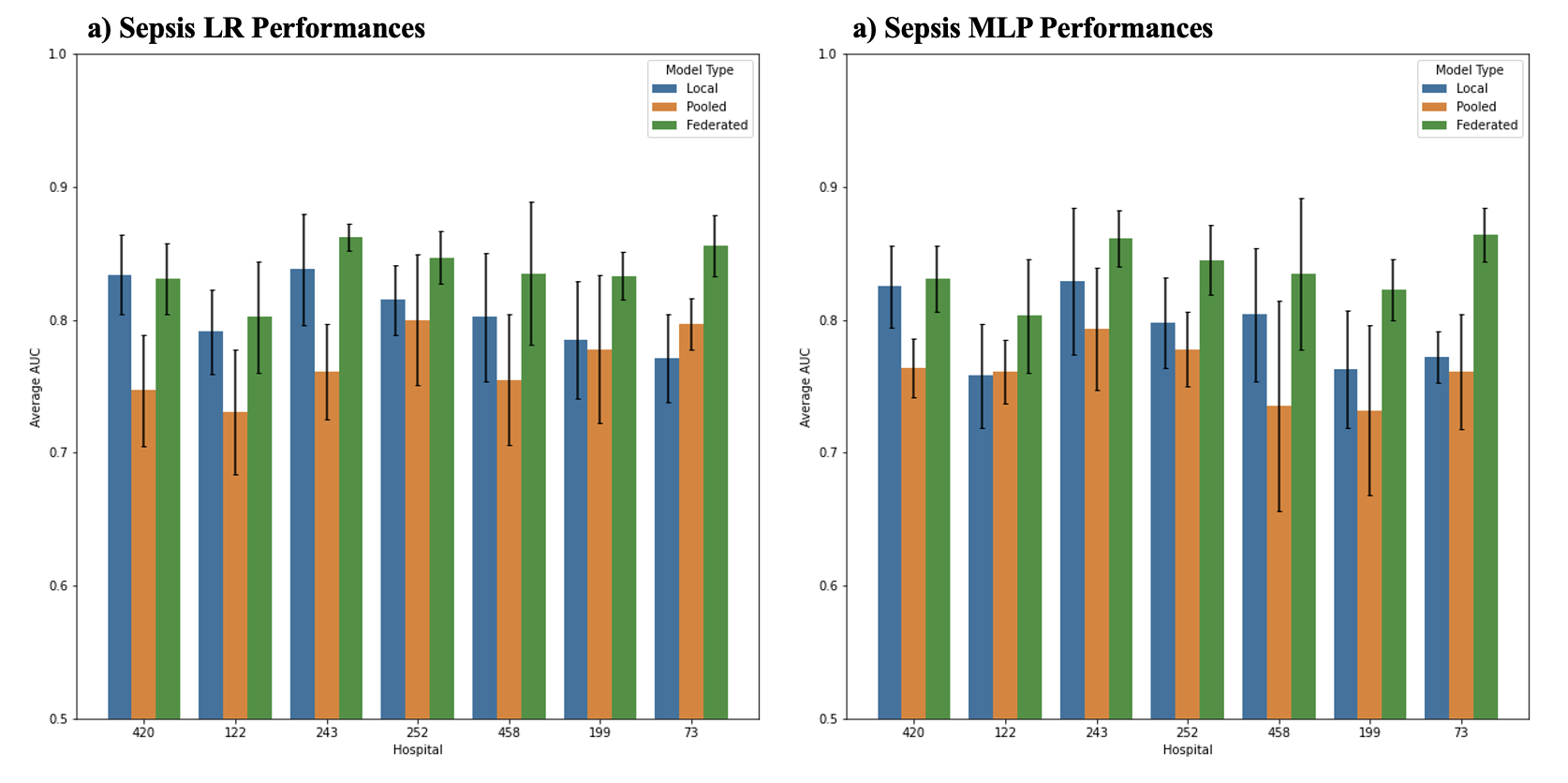

Supplement: S6 Fig — Each plot shows performances for the sepsis prediction setting. Blue bars depict each local site’s model performance on their respective site test data. Orange bars depict pooled model performance on each local site’s test data. Green bars depict federated model performance on each local site’s test data. (TIF) [file pdig.0000117.s009.tif]

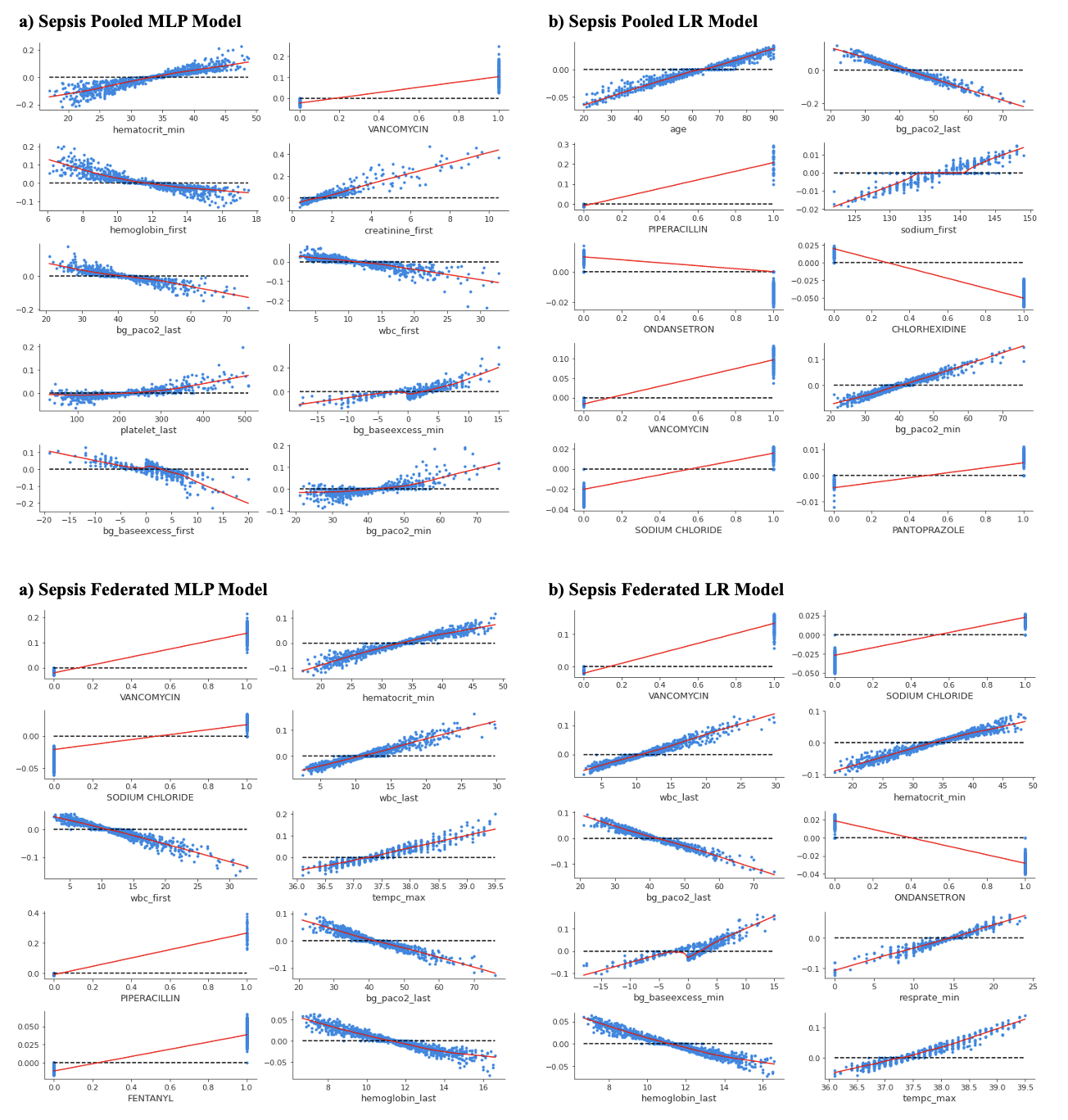

Supplement: S7 Fig — Each panel shows the marginal effects of each of the most impactful features ranked among the top 10 for predicting sepsis using pooled and federated models. The x-axis gives the raw values of each feature, and the y-axis gives the logarithmic of estimated odds ratio (i.e., the SHAP value) for sepsis when a feature takes a certain value. Each dot represents the SHAP value of a sample. The LOWESS curve, used for smoother extrapolating across all the dots, is plotted in red for all panels. (a, c) show Shapley dependence plots for MLP models and (b, d) show Shapley dependence plots for LR models. (a, b) show plots for pooled models, (c, d) show plots for federated models. (TIF) [file pdig.0000117.s010.tif]

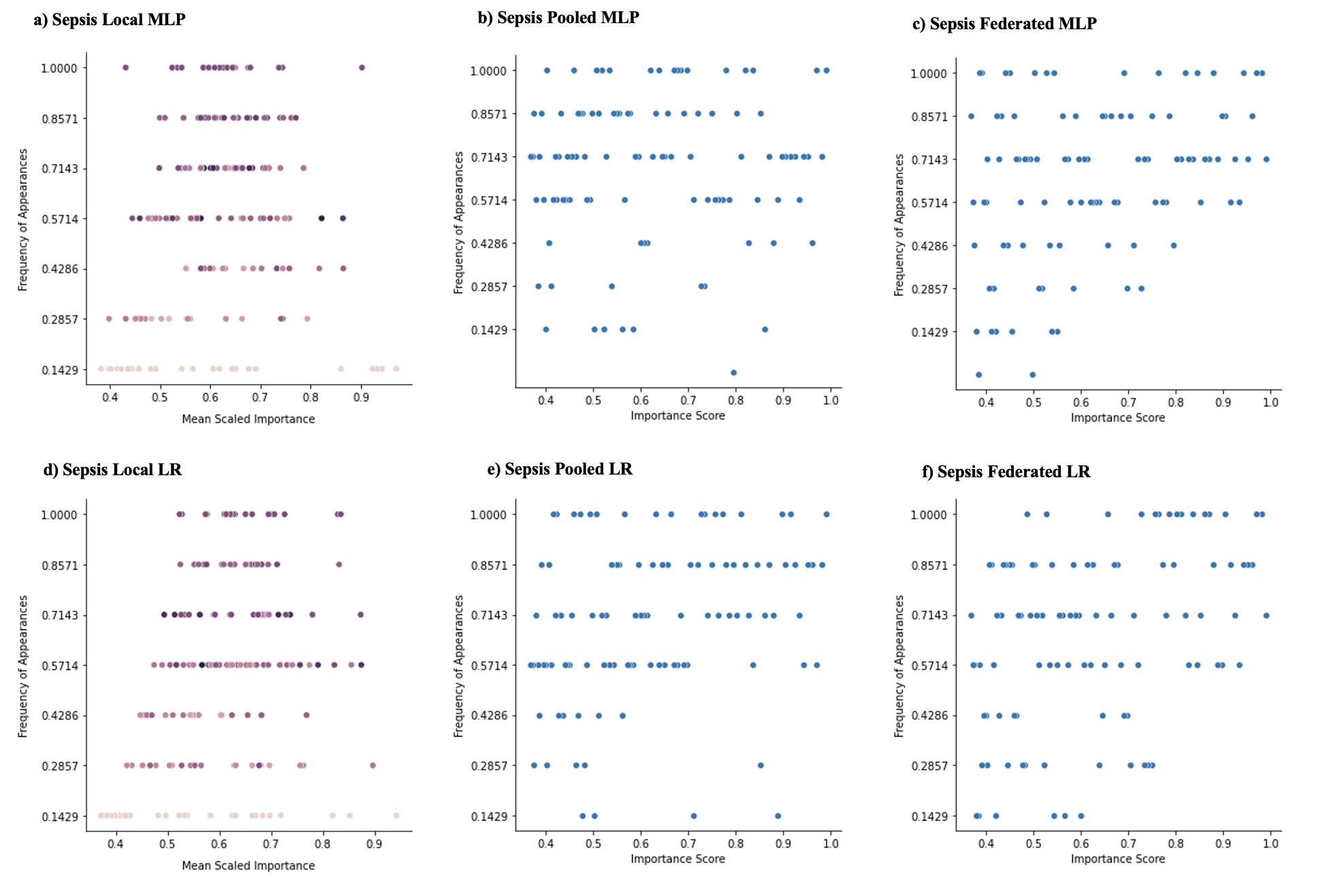

Supplement: S8 Fig — The figure demonstrates feature importance disparities for the sepsis setting and model architectures (MLP and LR). (a-c) show feature importance disparities for MLP models. (d-f) show feature importances for LR models. Each dot corresponds to one of the most important features ranked among the top-100 by at least one of the seven models; y-axis measures the proportions of sites that identified the feature as top-100, or “commonality across sites”; x-axis measures the mean of feature importance rankings measured as “soft ranking” (the closer it is to 1, the higher the feature ranks). Top-100 is an arbitrary cutoff we used to analyze the most important features to illustrate heterogeneity. In (a, d) each feature is also color coded by the interquartile range (IQR) of the ranks across sites (the higher the IQR is, the more disagreement across sites on the importance of that feature). (b, e) show the most important features for the pooled models. (c, f) show the most important features for the federated models. (TIF) [file pdig.0000117.s011.tif]

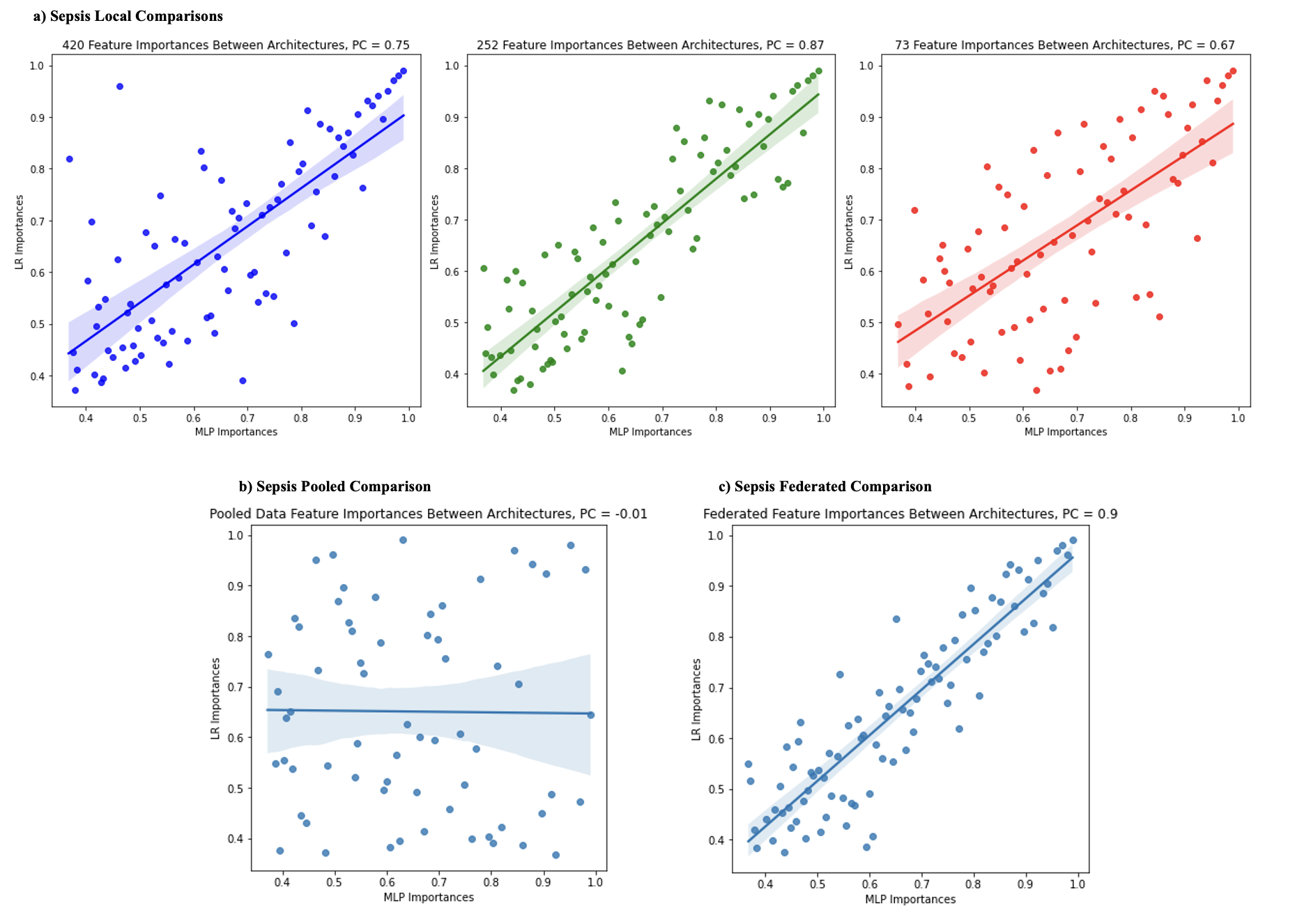

Supplement: S9 Fig — The figure shows correlations between important features in the MLP and LR models. Each dot corresponds to one of the most important features ranked among the top-100 by both the MLP and LR model. The y-axis measures the importance of the feature in the LR model whereas the x-axis measures the importance in the MLP model. The shaded portion represents a 95% confidence interval. PC (Pearson correlation coefficient) for each comparison is denoted on the top-left of each plot. (a) shows the comparisons for local sites, 420, 252, and 73. (b) shows comparisons for the pooled models. (c) shows comparisons for the federated models. (TIF) [file pdig.0000117.s012.tif]
