# Supplementary material for: Data heterogeneity in federated learning with Electronic Health Records: Case studies of risk prediction for acute kidney injury and sepsis diseases in critical care
Source: PLOS Digit Health. 2023 Mar 15;2(3):e0000117. doi: 10.1371/journal.pdig.0000117 (PMC10016691; doi:10.1371/journal.pdig.0000117)
Supplement: S1 Text — (DOCX) [file pdig.0000117.s013.docx]

**S1. Abbreviations**

| EHR | electronic health records |
| --- | --- |
| FL | federated learning |
| AKI | acute kidney injury |
| ICU | Intensive Care Units |
| AI | artificial intelligence |
| ML | machine learning |
| PHI | protected health information |
| MLP | multilayer perceptron |
| LR | logistic regression |
| ANN | artificial neural network |
| AUC | area-under-receiver-operator-curve |
| SHAP | Shapley Additive exPlanations |
| UIS | unique importance score |
| PC | Pearson-correlation coefficients |
| CTICU | Cardiothoracic Intensive Care Units |
| SICU | Surgical Intensive Care Units |
| CCU-CTICU | Critical Care Cardiothoracic Intensive Units |
| MICU | Cardiothoracic Intensive Care Units |
| SCU | special care unit |
| OW | observation window |
| SCr | serum creatinine |
| PW | prediction window |
| MICE | Multiple Imputation by Chained Equations |
| LOWESS | Locally Weighted Scatterplot Smoothing |
