# Supplementary material for: Data heterogeneity in federated learning with Electronic Health Records: Case studies of risk prediction for acute kidney injury and sepsis diseases in critical care
Source: PLOS Digit Health. 2023 Mar 15;2(3):e0000117. doi: 10.1371/journal.pdig.0000117 (PMC10016691; doi:10.1371/journal.pdig.0000117)
Supplement: S2 Text — (DOCX) [file pdig.0000117.s014.docx]

**S2. Validation on Sepsis Prediction Setting**

**Development and validation of Sepsis prediction models in local sites.** Local sepsis prediction LR models' performances ranged between 0.771 - 0.834 across sites, whereas MLP models' performances ranged between 0.772 - 0.829. Pooled prediction LR models' performances ranged between 0.731 - 0.800 across sites, whereas MLP models' performances ranged between 0.732 - 0.793. Pooled LR models significantly underperformed the local models at sites 420 and 243 (p < 0.01). Pooled prediction LR models' performances ranged between 0.731 - 0.800 across sites, whereas MLP models' performances ranged between 0.732 - 0.793. Pooled MLP model significantly underperformed the local model unique to site 420 (p < 0.01). Federated LR models' performances ranged between 0.833 - 0.862 across sites, whereas MLP models' performances ranged between 0.823 - 0.861. Federated LR models significantly overperformed the local model at site 73 (p < 0.01). Like the federated LR model, the MLP model significantly overperformed the local model unique to site 73 (p < 0.01) (S6 Fig).

**Clinical interpretation of sepsis and AKI prediction models in health systems.** The pooled MLP model identified minimum hematocrit level (*hematocrit_min*), Vancomycin, first measured level of hemoglobin (*hemoglobin_first*), first measured level of creatinine (*creatinine_first*), last measured partial pressure of carbon dioxide (*bg_paco2_last*), first measured white blood cell level (*wbc_first*), last measured platelet count (*platlet_last*), minimum pH value of blood (*bg_baseexcess_min*), first measurement of pH value of blood (*bg_baseexcess_first*), minimum partial pressure of carbon dioxide (*bg_paco2_min*) as top 10 important variables. All except Vancomycin are lab tests or vital sign measurements. The pooled LR model found numerous medications important in addition to Vancomycin, including Piperacillin, Ondansteron, Sodium Chloride, Chlorhexidine, Pantoprazole. Among these factors, hematocrit_min, hemoglobin_first, creatinine_first, bg_paco2_last, wbc_first, platlet_last, bg_baseexcess_min show slightly non-linear relationship with sepsis risk. In particular, a last measured platelet count (*platlet_last*) > 300 K/mcL is associated with greater sepsis risk. Minimum hematocrit levels (*hematocrit_min*) > 40% increases the odds ratio of sepsis by exp(0.1) = 1.1-fold. Exposure to vancomycin medications increases the odds ratio of sepsis by exp(0.1) = 1.1-fold as well. Most of the important factors in the LR models show a linear relationship with the risk of sepsis. An age of greater than 60 is associated with increased risk of sepsis. Administration of vancomycin is also associated with an exp(0.1) = 1.1-fold increase in risk of sepsis. Administration of piperacillin is associated with an exp(0.2) = 1.2-fold increase in risk of sepsis.

The federated MLP model considered more medications as important compared to the pooled MLP model. These medications included Sodium Chloride, Piperacillin, and Fentanyl. Administration of all these medications is associated with an increased risk of sepsis. The federated LR model found minimum respiration rate (*resprate_min*) and maximum temperature (*tempc_max*) important factors as well. A maximum temperature > 37.5 °C is associated with an increased risk of sepsis.

**Feature importance heterogeneity across model architectures, frameworks, and sites.** S8 Fig shows the feature importance heterogeneity plots for the sepsis prediction setting. Medications such as Lispro (site 420), Ceftriaxone (site 420), Hydromorphone (site 73), Calcium Gluconate (site 73), and Albumin (site 73) were considered highly important at only one site by the MLP model. These high importance yet unique features are generally shared across model architectures (LR and MLP) although there were some discrepancies. Some features that are considered universally important across most, if not all, sites for sepsis include tempc_max, Sodium Chloride administration, tempc_first, bg_ph_last, and resprate_first. For the pooled MLP model, hematocrit_min, hemoglobin_first, albumin_min were highly important universal features. Interestingly, for the pooled LR model, a different set of features were considered highly important and universal across local sites: age, administration of Sodium Chloride, and glucose_last. The pooled MLP model has a feature (platlet_min) which is uniquely important to the pooled model (i.e., these features were not considered as part of the top 100 features at any local site). This feature is moderately important to the pooled MLP model.

Like the AKI settings, both MLP and LR pooled models have relatively fewer features that are only important at a small number of sites compared to the local model framework. For the pooled MLP model, hematocrit_min, hemoglobin_first, albumin_min were highly important universal features. Interestingly, for the pooled LR model, a different set of features were considered highly important and universal across local sites: age, administration of Sodium Chloride, and glucose_last. The pooled MLP model has a feature (platlet_min) which is uniquely important to the pooled model (i.e., these features were not considered as part of the top 100 features at any local site). This feature is moderately important to the pooled MLP model.

For the federated MLP model, hematocrit_min, administration of Sodium Chloride, and tempc_max was among the highly important universal features. The federated LR model shared some of its important features with the MLP but also included others like wbc_last, heartrate_first, and age. While the differences between federated LR and MLP models weren't as pronounced as they were in the pooled framework, there are discrepancies. Like the pooled MLP model, the federated MLP model has features uniquely important to it, including platelet_min and administration of Dexamethasone.

**Correlation of feature importances across model architectures.** S9 Fig shows cross-architecture correlations for the sepsis setting. Local models have a moderately strong positive correlation between MLP and LR feature importances with Pearson-correlation coefficients (PC) ranging from 0.67 - 0.87 depending on the site. For the pooled models, there is no significant correlation (PC = -0.1) between MLP and LR models, suggesting that the pooled MLP and LR models have a lot of disagreement on the importance of features. The federated model has a strong positive correlation between the two model architectures, with the PC being significantly higher than any local site analysis. This suggests that, within the sepsis setting, the federated framework was successful at decreasing feature discrepancies between the LR and MLP architectures that were present in local models.
